# Experimental study on the parameters affecting the performance of spiral tube pump

**DOI:** 10.1038/s41598-024-73234-w

**Published:** 2024-09-28

**Authors:** Million Meseret, Dawit Wami, Ramesh Babu Nallamothu

**Affiliations:** 1 Department of Mechanical Engineering, College of Engineering, Assosa University, Assosa, Ethiopia; 2 Department of Mechanical Engineering, School of Mechanical Chemical and Materials Engineering, Adama Science and Technology University, Adama, Ethiopia

**Keywords:** Spiral tube pump system, Manometric pump, Experimental investigation, Important factors, Energy science and technology, Engineering

## Abstract

This experimental study delves into the critical parameters influencing the performance of spiral tube pumps—a centuries-old technology attributed to H.A. Wirtz in 1746. The Spiral Tube Pump system, constructed using a transparent flexible tube wound spirally on a waterwheel, harnesses the kinetic energy of a river, offering a simple, cost-effective, and emission-free irrigation solution using locally available materials. Operating on the principles of a manometer, water and air plugs are pushed into the pipe alternately by the pump. Key factors crucial to enhancing the performance of this technology are investigated, focusing on submergence ratio, rotational speed, outer diameter, and the number of turns of the spiral tube. The experimental setup involves a prototype featuring a 4-meter-long, ¾-inch diameter transparent flexible tube spirally wound on a 1.5-meter-wide water wheel, achieving a maximum output of approximately 30 L per minute discharge and a 3.3-meter head. Through a series of rigorous experimental procedures, it becomes evident that wheel speed and submergence ratio significantly impact the pump’s discharge. For 600% increase in wheel speed, up to about 500% discharge increase was observed. Also for 300% increase in submergence ratio, up to about 275% increase in discharge was observed. The number of spiral turns predominantly influences the pump’s head. For 100% increase in spiral turn, up to about 33% increment in head was observed. And the outer diameter affects both discharge and head. For 87% increase in outer diameter, up to about 80% of increase in discharge and up to about 163% increase in head was observed. These findings underscore the adaptability of the Spiral Tube Pump design to specific irrigation field conditions. For instance, in scenarios where the irrigation field is elevated relative to the river, incorporating a greater number of spiral tube turns can enhance the pump’s efficiency in transporting water to the desired location.

## Introduction

The spiral tube pump, an ancient technology developed in 1746 by H.A. Wirtz in Zurich, Switzerland, is hailed as a cost-efficient water pump powered by renewable energy^1–3^. It can be constructed from locally available materials and repaired by local craftsmen. It can works by integrating with another ancient technology, the water wheel. While the water wheel harness the kinetic energy of the stream the spiral tube pump will deliver portion of the stream to the irrigation field. Even if it may not match the output of conventional pumps, its sustainability, simplicity, and local manufacturability make it a viable solution for regions with abundant river water resources, such as Ethiopia.

**Fig. 1:**
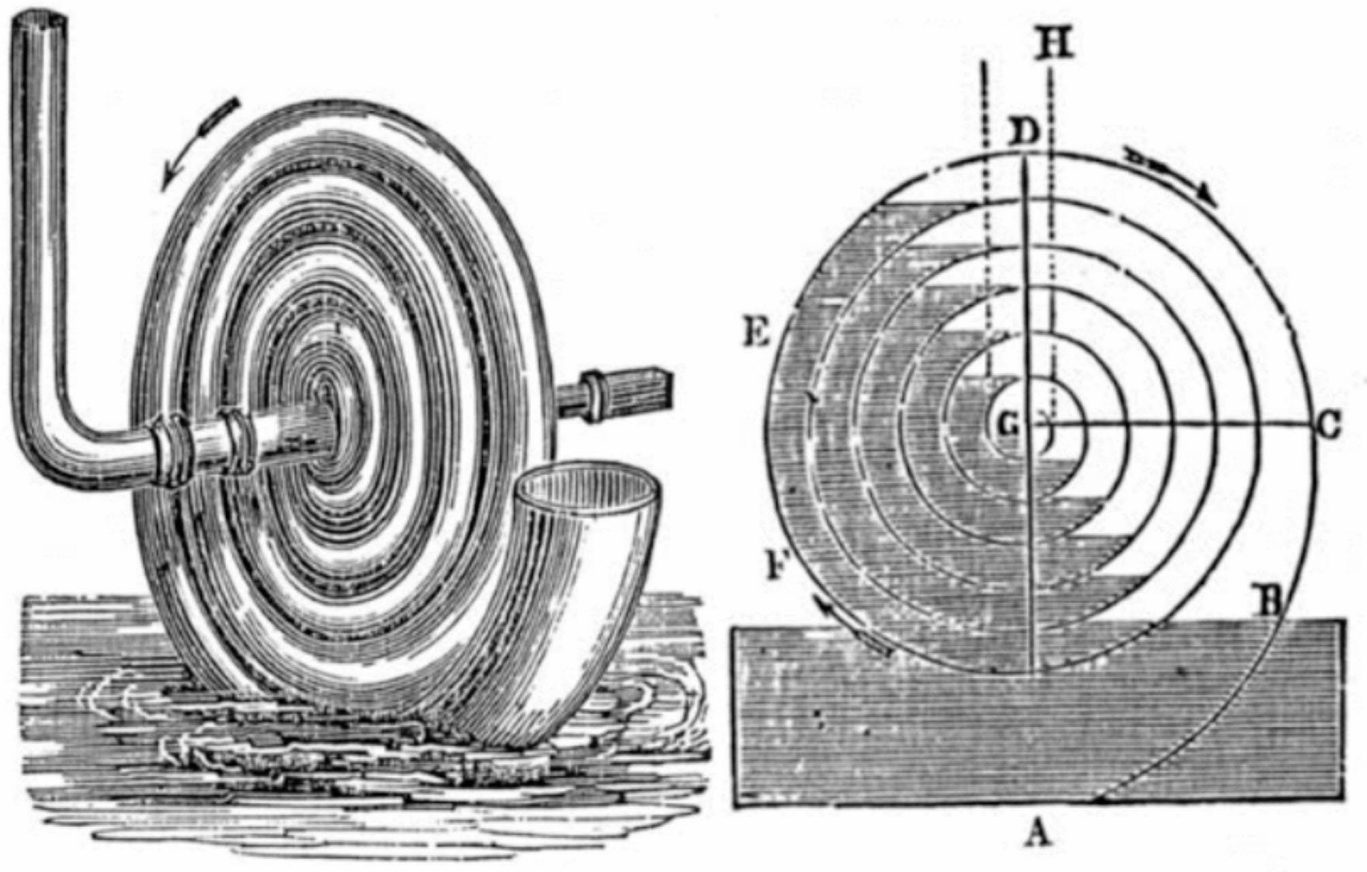
Historic Wirtz pump − 1842 drawing^1^.

Even though it is an invention that has been around, for a while there hasn’t been much literature about its performance. Considering its relevance in the context of developing countries it is important to investigate and understand its potential. Different names have been used to refer to this pump, such as Wirtz pump, Hydrostatic pump, Coil pump and Manometric pump each indicating orientations and geometries within the pump system^3^. Figure 1 depicts historical Writz pump.

A Spiral Tube Pump is comprised of a spirally turned tube around a drum or disk’s exterior or interior that is partially submerged in water and has its axis parallel to the water’s surface. The tube has two ends: an open end that is fixed to the drum and acts as the inlet, and a sealed rotary joint that joins the other end to the delivery pipe. The length ratio is dictated by the immersion depth of the drum, and as the drum rotates, the inlet end of the tube receives alternating plugs of water and air^1^. These plugs move down the helical pipe to the outlet, then up the delivery pipe to the header tank after going through the rotary joint.

The pressure required to push the plugs up the delivery pipe is created because the water plugs acts as a manometer maintaining the pressure difference across it. The sum of these pressure variations contributes to the pumping effect^4^. In this way the Spiral Tube Pump efficiently uses the wheels rotation to establish a pressure gradient facilitating the upward movement of air and water plugs for effective water transportation.

## The working principle of spiral tube pumps

The “Spiral Tube Pump,” sometimes referred to as the “coil pump” or the “manometric pump,” functions according to the same principles as a manometer, advice for measuring liquid pressure, including air and gases. A manometer works on the principle of hydrostatic equilibrium to gauge the static pressure that a stationary liquid or gas is exerting^4,5^. According to hydrostatic equilibrium, the pressure at any point in a fluid at stationary determined by the fluid layer’s weight. Basically a manometer usually comprises of an incompressible fluid, such water or mercury, contained in a U-shaped tube.

**Fig. 2:**
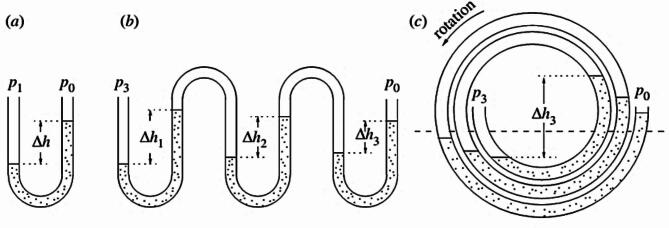
Working principle of spiral tube pump; (a) U shape manometer (b) Cascaded manometer (c) Spiral tube pump^5^.

Figure 2 provides a visual representation of functional principle of Spiral Tube Pump. In Fig. 2a, a U-tube manometer is shown, a simple pressure gauge based on Pascal’s Law, which states that the height difference (h) between the surfaces of the water in the right and left sides is proportional pressure difference (P1 − P0). Expanding upon this idea, Fig. 2b shows such manometers cascaded in series, leading to a rise in pressure difference (P3 − P0) proportional to the sum of the heights (h1 + h2 + h3). Finally, Fig. 2c envisions the vertical concept in spiral configuration, with the pressure difference being proportional to the total of the height differences^1,3,5^.This conceptual explanation helps illustrate how the Spiral Tube Pump utilizes the principles of manometer and Pascal’s Law to generate a cumulative pressure difference, enabling its efficient operation.

To put it briefly, the pump forces alternating plugs of air and water into the pipe with each turn. The air in the pipe is compressed as the wheel rotates, creating a pressure head inside each coil. A total head is built-up at the inner coil, forcing the water out of the delivery pipe. This caused each coil’s water to be displaced, creating a pressure head. Accordingly, one may say that the Spiral Tube Pump to be a sort of “positive displacement pump”^6^.

The two main types of pumps that are examined in the literatures are Spiral Tube Pumps, which are essentially planar arrangements in which the radius of successive turns decreases, and coil pumps, in which the pipe is formed into a series of turns with the same radius (the pipe may be imagined to be wound around a cylinder helically). But based on their working principle we can name both of them as manometric pump, following their likeness to an injured cascading manometer. These various forms give rise to manometric pumps, which are referred to by a number of names in the literature and are occasionally used synonymously. However, for the purposes of this work, Spiral Tube Pump (SP), coil pump (CP), and conical helix pump (CHP) will be the terms used to refer to planar cross-flow, non-planar cross-flow, and axial-flow non-planar pipes, respectively^8^.

## Literature review of significant studies and essential theoretical relations of important parameters

The literatures on Spiral Tube Pumps and related manometric pump systems are predominantly dated, reflecting a decline in interest in developed countries with the emergence of conventional electric and fuel pumps, with high output and widely adopted. However, the recent focus in developing countries on seeking affordable pumping systems has revived interest in Spiral Tube Pumps and similar alternatives. Although the available literature may not be recent, several foundational works are crucial for comprehending manometric pumps, providing essential insights into their overall understanding and findings. These seminal studies remain significant, as limited research has been conducted in this field in recent years. Despite their age, these key literature sources continue to serve as essential references for gaining a comprehensive understanding of manometric pumps.

**Fig. 3:**
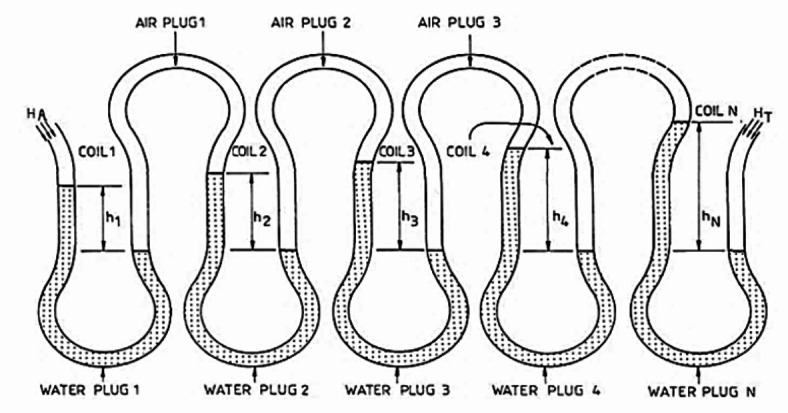
Cascaded manometer^3^.

Among few literatures the work of G.H. Mortimer & R. Annable called “The Coil Pump – Theory and Practice” is the one which explain the principle of coil pump well with the help of analytical relationships^3^. In this work, a basic pump was created, and via research in the lab, a theory that adequately predicts the behavior of the pump was constructed. The pressure developed in the pump is represented by a cascaded manometer. As a cascade manometer it develops the pressure head difference across the coil pump when it is rotating or stationary. And the total head difference throughout the manometer is equal to the total of the head variations throughout the water plugs, this is shown in Fig. 3.

1 $$H_{T} - H_{A} = h_{1} + h_{2} + h_{3} + \cdots h_{N}$$

where *H*_*T*_ is the outlet’s absolute pressure head, *H*_*A*_ is the atmospheric head, and N is the number spiral turns.

The important concept in manometric is the compression of the air plugs that cause the pressure difference. According this literature for calculation on the length of the plugs *P.V*^*1.15*^*= constant* is valid, where p is the air plug’s absolute pressure, and V is the plug’s volume. Considering that the coil’s pipe diameter remains constant:

2 $$H_{n} L_{A}^{{1.15}} = H_{n} L_{{A.n}}^{{1.15}}$$

where LA is length of air plugs. In pump performance study the other important factor is the pump discharge. In this literature the researcher had stated a clear theoretical relationship to predict the steady pump discharge. That is:

3 $${\text{Q}}_{{\mathrm{p}}} = {\text{N}}_{{\mathrm{s}}} {\uppi}\,{\text{r}}^{2} .{\text{L}}_{{{\text{w}}.1}}$$

where r is the helical pipe’s diameter and _0.1_ is the amount of water plug inserted into the inlet. Without any dynamic losses .1 = 1. . Where 1 is the angle the water plug sustains. When the flow rate was 0.80 m per second. Even at 0.40 m/s stream velocity, the pump was operating at a diminished performance.

In 2005, Sadek Z. Kassab conducted a noteworthy experimental research study on the coil pump in Egypt^8^. The primary objective of this study was to evaluate the coil pump’s performance under various operating parameters, with a specific focus on rotational speed, submerged ratios and the number of coils in the wrapped hose.

The investigation delved into the impact of pump rotational speed, variances in the submerged ratio, and changes in the number of coils to gain a comprehensive understanding of the coil pump’s performance. The experimental results were meticulously compared with theoretical outcomes derived from Mortimer and Annable relationships, revealing a noteworthy alignment, particularly at lower rotational speeds. Also the experimental results were compared with the theoretical results from relationships Mortimer and Annable, and Good agreement had been obtained. Especially for lower rotational speeds.

In conclusion this report the following major points are stated;

1. Depending on the working submerged ratio and design parameters, raising the pump’s rotational speed raises the water flow rate until it hits its maximum, at which point increasing the rotational speed decreases the discharge. .
2. The maximum static head of the pump is not significantly affected by increasing the pump’s spinning speed. .
3. Increasing the submerged ratio causes the pump flow rate to climb until it reaches its maximum, which is dependent on the pump’s rotating speed. Once the pump is entirely submerged, the flow rate reduces to zero, regardless of the pump’s speed. .
4. The maximum static head is slightly impacted by the submerged ratio; it is almost constant until the pump submerged ratio hits 100%, at which point it sharply drops to zero. Increase number of coils increases the pump head, while pump discharge is nearly constant.

Peter Tailer discussed very important concepts other than his experimental journey such as; Flow over, Blow-back, and Air Lift. In addition to this he devised an equation for number of turns^1^. A theoretical relationship is presented to find h_n_ (head in n-the coil) and n (number of coil) with given value of H (delivery head), D (outer coil diameter) and d (pipe diameter) based on Boyle’s low.

4 $$n=2 H/(D+{h}_{n})$$

The most complete in enable engineers to design and implement the Spiral Tube Pump. Ludwig C.A. Naegel in Munich, 1998 designed, constructed and tested Spiral Tube Pump locally^2^. Naegel described Spiral Tube Pump as economical water pumps that are constructed using locally accessible materials, run on renewable energy, and can be fixed by skilled local craftsman. After serious of careful testes, multiple linear and non-linear regression analysis was used to determine the impact of each variation on the pump’s performance. In nearly 1,600 individual test runs, the maximum static head is described by sum of coil diameters (*scd*),

5 $$max.\:H\left(m\right)=0.97scd$$

According to the given literatures the following parameter affects the performance of a manometric pump in general. A serious experimental investigation has been conducted on coil pump types. Those from existing literatures it is not clear that how those parameters affect Spiral Tube Pumps. Though, since both coil and Spiral Tube Pumps share same working principle it is clear that have similar performance affecting parameters.

1. ***Spiral tube outer diameter***: The head created increases in proportion to the spiral turns’ outer diameter^9^.
2. ***Number of tube turns***: A coil pump’s number of tube turns is directly correlated with the head it generates^9^. As per Kassab, one can assess the efficacy of a coil pump by manipulating its number of coils, submerged ratio, and rotational speed (N). They discovered that while the discharge (QP) was constant, the pressure head (HP) rose as the number of coils grew^8^ .
3. **Tube diameter**: As reported by Mortimer and Annabel the spiral tube diameter doesn’t affect the pressure head. Its effect is related to the pump discharge^3^.
4. ***Submergence ratio (Sr)***: Discharge rises with submerged ratio but has no impact on head^8^. Discharge will be zero when either the pump is fully immersed or completely out of the water.
5. ***Wheel velocity (N)***: While the maximum static head obtained is not much affected by an increase in rotational speed, discharge increases with an increase in rotational speed until it reaches its maximum value, depending on the working submerged ratio. At a certain maximum speed and submerged ratio, discharge goes to zero^8^.

6. ***Stream flow velocity***: The water’s speed and the water wheel’s rotating speed are exactly proportionate^9^.
7. ***Number of spiral layers***: According to N.R Patila (2013) study of two layers of spiral tube and found that for double layer, discharge is higher than the single layer at all rotational speeds^10^.

Among the seven parameters, tube diameter, stream flow velocity, and number of spiral layers were excluded from this study, leaving four parameters to be examined. The effects of tube diameter on fluid flow are well-documented in existing literature. Stream flow velocity is accounted for by wheel velocity, as it primarily influences rotational speed. As for the number of spiral layers, understanding the performance of a single layer allows for extrapolation to multiple layers, enabling the determination of cumulative performance.

There for this study focuses on examining the effect of important parameter: submergence ratio, rotational speed, outer diameter, and the number of turn on the performance of the Spiral Tube Pump. So its main objective was to conduct experiments that examine the effects of the submergence ratio, rotational speed, outer diameter, and the number of turns in the performance of spiral tube pump.

### Motivation of the study

Recently in Ethiopia having adequate food becomes harder due to the inflation. This may have both international and local reasons though the solution mainly depends on the increasing of productivity of the agricultural sector. The productivity of this sector mainly hangs on the efficient management of water resource which implies the effective irrigation that enables production more than once in a year. Moreover currently the government policy focus in production of vegetables, fruit and wheat to make the country self-sufficient on feeding the citizen and establishing social stability. Realising such policy without cultivating the water resources of the country effectively is impossible. In such situation a Spiral Tube Pump system is a viable option because it can be manufactured using locally available materials and workshops. Additionally, it is emission-free, has low manufacturing and operating costs, and offers reasonable performance. So If the knowledge gap in this technology is addressed and its performance is maximized and optimized, the Spiral Tube Pump system can become a valuable alternative to support agriculture in developing countries, ensuring food security for every citizen and their families.

### Contributions of the study

The major contributions of the study are:

- It introduced the technology to university community and the broader society, providing a comprehensive overview of its working principles and potential applications.
- It demonstrates that the Spiral Tube Pump can be manufactured using locally available materials and workshop resources, promoting self-sufficiency and cost-effectiveness.
- It identifies the strengths and weaknesses of the Spiral Tube Pump system, offering a balanced assessment that can inform future improvements and applications.
- Mainly the study identifies and examines important parameters that are affecting the pumps performance to provide insights into optimizing the system for better efficiency and reliability.

### Organization of the paper

This study report is organized into four main sections; Introduction, Methodology, Result and Discussion and Conclusion, and with several subsections. The Introduction provides an overview of spiral tube pump technology, its historical context, and relevance including a Literature Review discussing previous studies and theoretical principles related to spiral and coil pumps. The Methodology details describe the experimental prototype, setup, design and the procedure including relevant information to duplicate the process. The “Results and Discussion” section presents and analyses the experimental findings on the effects of submergence ratio, rotational speed, outer diameter, and the number of spiral tube turns on pump performance. The Conclusion summarizes key findings, offer optimization suggestions, and propose areas for future research. At last it lists the references cited.

## Methodology

### Experimental prototype

For an in-depth investigation into the parameters influencing the performance of the spiral tube pumping system, a prototype has been meticulously designed and crafted. This prototype involves the use of a 4-meter-long, ¾-inch diameter transparent flexible tube, expertly wound in a spiral pattern on a 1.5-meter-wide water wheel. The spiral tube is intricately attached to the delivery tube through a locally fabricated rotary joint. It is important to note that the joint exhibited some leakage, a factor duly considered during the subsequent analysis of the results. This leakage includes the escape of compressed air, which is essential for pressure build-up in the pump. Measuring the amount of escaped air was not possible due to the lack of appropriate measuring instruments and nature of the experiment. However, the amount of water leakage was estimated to be between 6% and 11% of the total discharge.

The experimental site selected for this study is the Kito River in Jimma town, providing a practical and real-world environment for conducting the experiments. The transparency of the flexible tube allows for a visual assessment of the pumping mechanism, facilitating a comprehensive understanding of the system’s behaviour under varying conditions at the chosen site.

### Design of experiment

Most importantly how submergence ratio, rotational speed, outer diameter and number of turns of the spiral tube affects head and discharge is studied. So that submergence ratio (Sr), wheel speed (w), outer diameter (Do) and number of turns of the spiral tube (n) are independent variables. Whereas Discharge (Q) and Head (H) are dependent variables. To continue experimenting sample value must be selected for each independent variables or factors. These sample values are stated in the Table 1.

**Table 1:** Study variables and levels.

Factors	Levels			
Wheel speed (RPM)	5	15	25	35
Submergence ratio	20%	40%	50%	80%
Number of spiral turns	3	4	5	6
Outer diameter	0.8	1	1.2	1.5

Those levels are selected based on reality or applicability. Meaning the wheel speed maximum level is selected to be 35RPM because in the experiment the wheel was rotated using human power. Based on several trial it observed that the maximum stable wheel speed that can be attained by rotating the wheel with hand is 35RPM. Also with lower stream speed the wheel speed can get slower up to 5RPM and much less.

Regarding to submergence ratio selection of submergence value is based on the depth of river and the water proof characteristics of the bearing. If the bearing is sealed and rust resistant we can immerse the submersion above 50% unless it is advised to immerse the pump near to 50% at maximum.

The number of Spiral Tube Pump implicates the weight of the water in the spiral tube if the spiral turn number is higher the mass of the system will rise as the wheel turns. So eventually it may stop rotating. Also the depth of the river limits the number of spiral turns.

The outer diameter is mainly restricted by the depth of the river. Most of Ethiopian rivers are mostly shallow for most of the season except in summer.

### Experimental setup

**Fig. 4:**
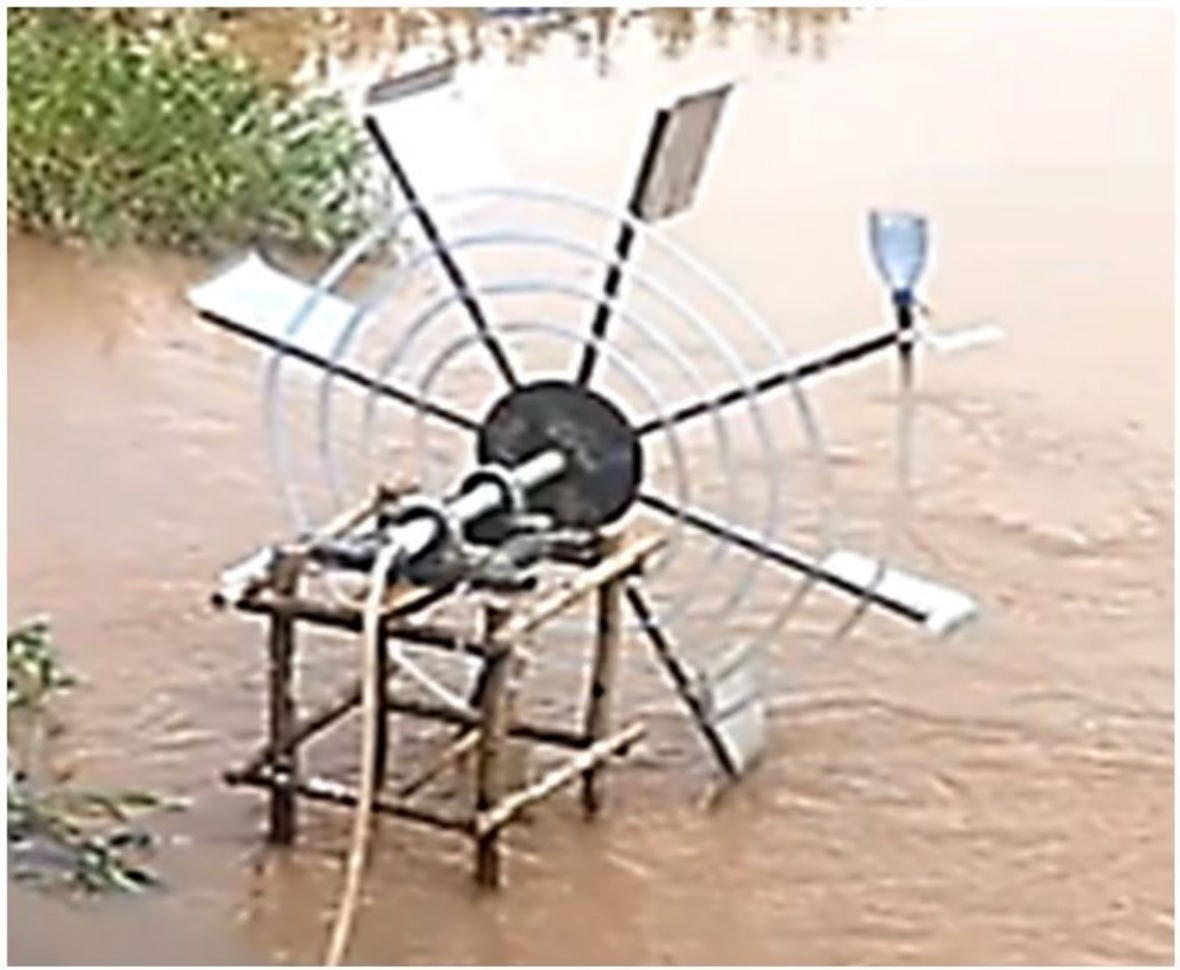
Experimental setup in Jimma town Kito River (Photo taken by the author).

The general experimental setup is shown on the Fig. 4. As shown the Spiral Tube Pump system consists both the spiral tube and a simple water wheel. The spiral tube is attached to the wheel using the metal wire. The pitch distant between each spiral turn is specified based on the number of turns. In the Fig. 4 there are 5 spiral turns with around 120 cm distance in between.

The whole system is placed over pre developed wooden structure planted firmly in the river. This structure has four levels. Those levels were suitable to place the stage on it. The primary purpose of these stage were to allow submergence ratio change. When the stage is placed on the top level the submergence level of the pump will be 20%, as the level decrease lower the submergence ratio will increase up to 80%.

The main objective of this experimental procedure is to study the effect of submergence ratio (Sr), Wheel speed (N), number of spiral turns (n), and Do on H and Q. in this experimental procedure the paddle was not needed, because it causes drag while rotating the wheel manually.

### Experimental procedure

Experiment is conducted using the following procedure;

1. First, the pump with the certain number of spiral turns is placed at certain level on the wooden structure. This determines the pump Submerged ratio (Sr).
2. The wheel is rotated manually with a certain speed. And this speed was recorded. This speed is the pump wheel speed (W).
3. The delivery pipe outlet was placed at pre-determined vertical distance from the river level. This vertical distance from the river level implies the pump vertical head (H) whenever there is discharge.
4. The pump discharge is collected in gallon bucket. Calculating a certain volume in the gallon bucket gives the pump discharge/flow rate (Q) at certain rotational speed, submergence ratio number spiral turns and head.
5. At the fifth procedure on experimental process was completed. The next will start by altering the number the number of spiral turns or by changing the submergence ratio.

In each step of the experimental procedure serious data recording and documentation was done.

### Uncertainty analysis

This subsection presents the uncertainty analysis performed to quantify the precision and reliability of the measurements taken in this study. The key sources of uncertainty are identified, quantified, and propagated to determine the overall uncertainty in the key performance metrics, namely discharge and head.

The primary sources of uncertainty in this study include:

- Measurement Errors: Inaccuracies in measuring discharge and head.
- Environmental Factors: Variations in environmental conditions, such as river flow variability and air condition.
- Human Errors: Variability introduced by manual operations and measurements.

Based on this the quantification and propagation of the uncertainties are stated as follows:

Quantification of Individual Uncertainties for the results:

**Discharge (Q)**:

- Measurement Error (σ_Q1_): ±0.5 L/min.
- River Flow Variability (σ_Q2_): ±0.2 L/min.
- Manual Measurement Variability (σ_Q3_): ±0.2 L/min.

**Head (H)**:

- Measurement Error (σ_H1_): ±0.1 m.
- Air Condition Variability (σ_H2_): ±0.05 m.
- Manual Measurement Variability (σ_H3_​): ±0.05 m.

Propagation of Uncertainties: To estimate the overall uncertainty in the key performance metrics, the individual uncertainties are propagated using the root sum of squares method. That is

6 $${\upsigma}=\sqrt{{{\upsigma}}_{1}+{{\upsigma}}_{2}+{{\upsigma}}_{3}}$$

Accordingly the propagated uncertainty will be σ_Q_ ​≈ 0.57 L/min for the discharge and σ_H_​ ≈ 0.12 m for the head. So based on the final result:

- **Discharge**: Q = 30 ± 0.57 L/min (at 95% confidence level).
- **Head**: H = 3.3 ± 0.12 m (at 95% confidence level).

This detailed uncertainty analysis provides a clear understanding of the precision and reliability of the experimental results, ensuring that the conclusions drawn from the study are based on robust and accurate measurements.

## Results and discussion

### Effect of important parameters on spiral tube pump performance

The effort to examine the effect of important parameters in the pump’s performance took more than 408 serious experimental procedures without including uncertain high number of trials. Each experimental procedure is repeated three times to preserve its certainty and avoid error as much as possible. Also they were much closer to the realistic working environment of the pump. Unlike previous studies the experimental procedure of this study is conducted in flowing river rather in laboratory in stationary tanker of water. The results of each experimental procedure are presented with graphs followed by the respective discussion.

### Effect of variation of wheel speed

The wheel speed is not parameter that can be controlled by the user in the real situation it is all dependent on the flow rate of the stream. Though since it is important to understand how it affects the pumps performance the variation in wheel speed is established by rotating the wheel manually with almost a constant speed. So the selected variations in wheel speed are in the range a wheel can be speed up manually with stability. So that after many trials 35 RPM is found to be the maximum stable speed that can be attained manually.

**Fig. 5:**
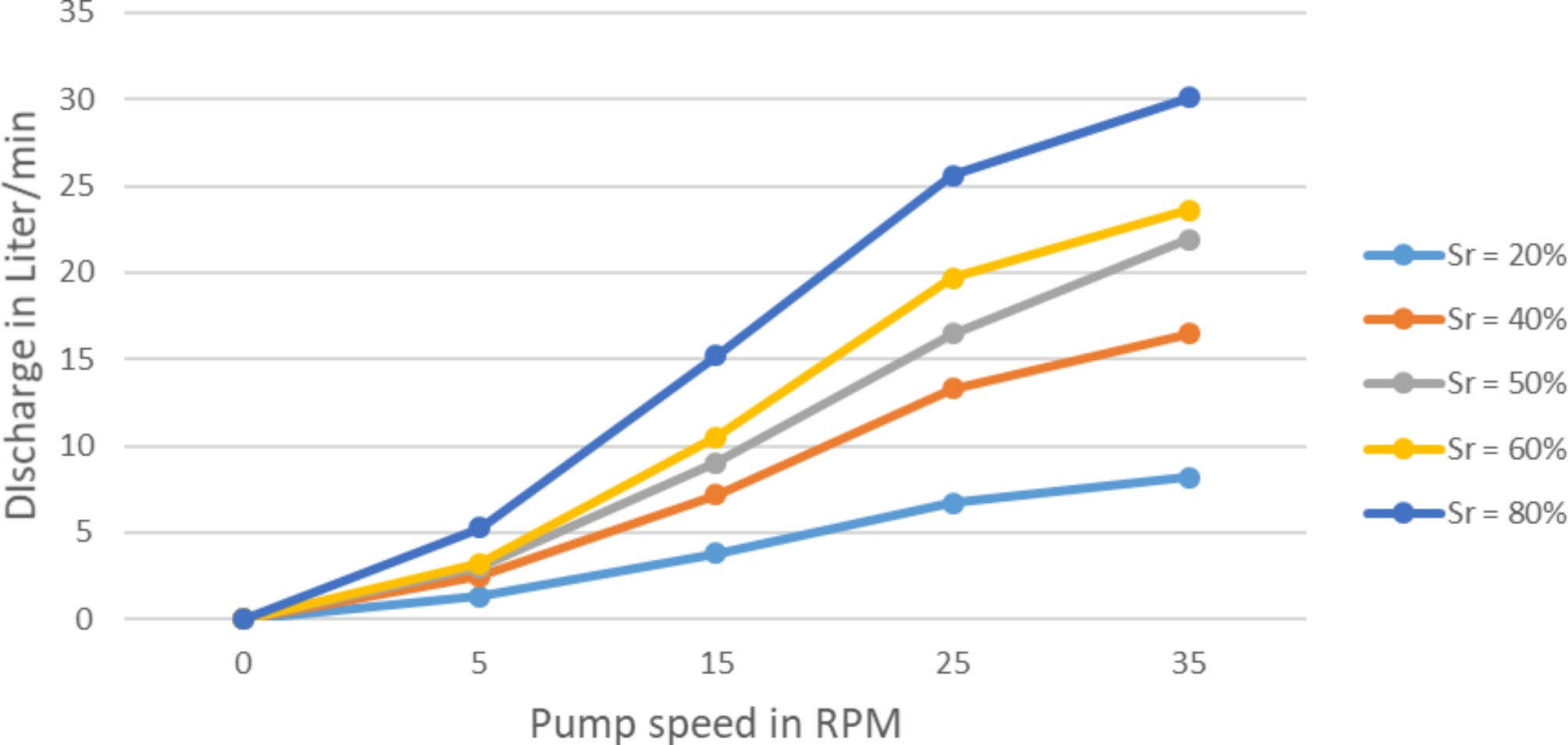
Pump discharge variation with rotational speed at various submerged ratios.

**Fig. 6:**
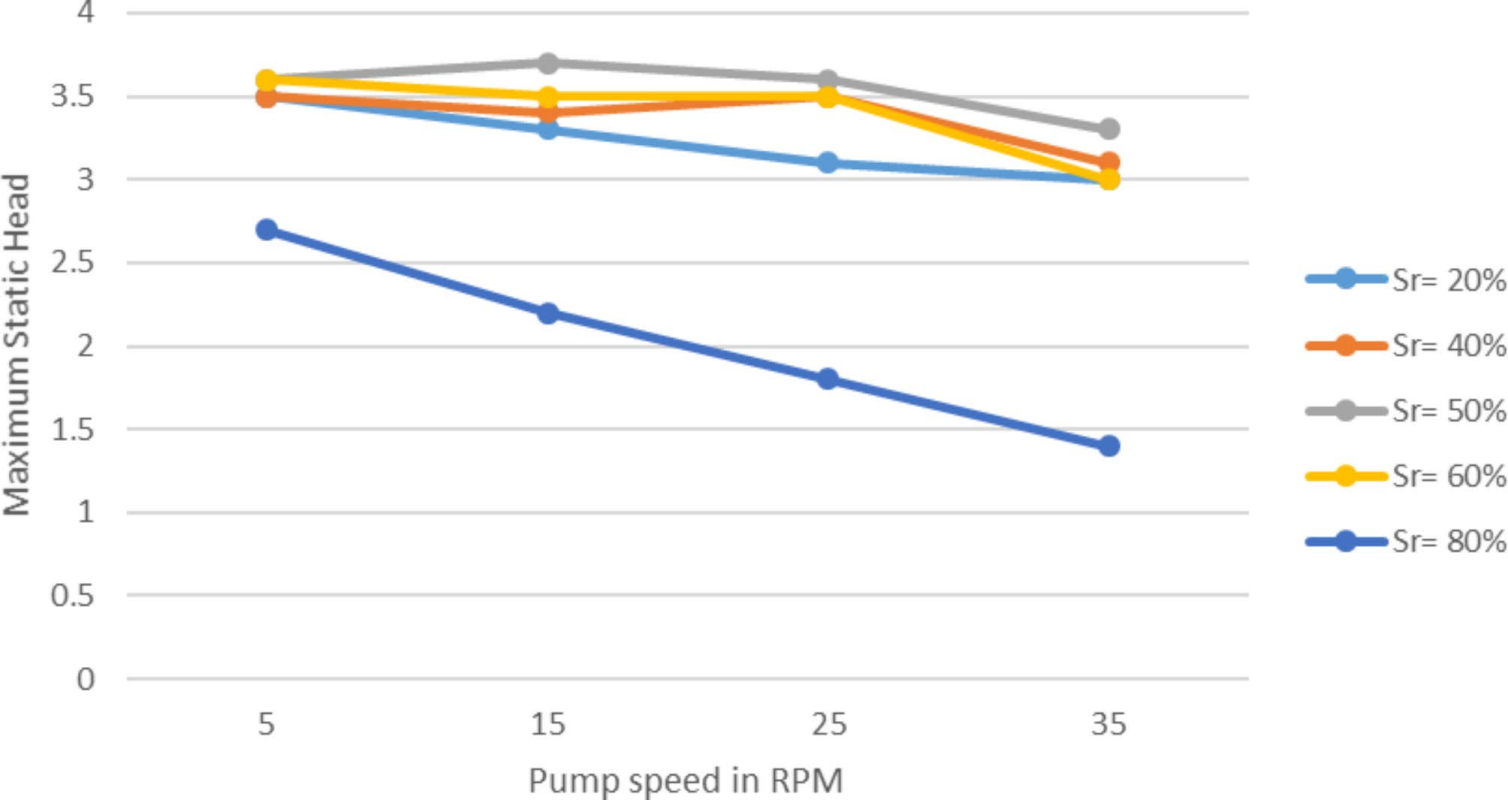
Max. Static head variation with rotational speed at various submerged ratios.

The impact of pump speed variation on the discharge (Q) and maximum static head (H) of the pump at different submerged ratios, Sr, as shown in Figs. 5 and 6 respectively. These results are for one layer, for outer diameter, Do = 1.5 m, tube diameter, dp = 3/4 in, number of spiral turns *n* = 5 turns and at head = 1.5 m. obviously with at zero RPM of rotation no output is expected. But when the rotational speed rises the discharge also rises proportionally. For instance on the 20% submergence ratio for 10 RPM increment in speed around 19 L per minute increment in discharge is exhibited. At the same submergence ratio with maximum speed of 35 RPM about 30 L of water is collected per minute. The discharge also showed a significant increase with the increase of submergence ratio. With 20% increase in Sr around 67% in discharge is observed. At maximum speed (35RPM) and with 80% immersion the pump yielded 200 L per minute which is astonishing.

This proves the theoretical relationship that stated by Mortimer and Annabel on equation 2.11 with 5% error. This means the variation of wheel speed affects the pump discharge directly and proportionally in similar manner as of the coil pumps. In other words while the wheel speed increase the Spiral Tube Pump discharge also increases. This may suggests the installation of the pump should be on location that the stream with higher velocity so that the pump wheel turns with the higher speed possible.

Unlike the discharge the maximum static head of the pump has shown almost no difference when the wheel speed is increasing. As a matter of fact it shown slight decrease at higher submergence, like on 80%. While the speed rises from 5 to 35 RPM the maximum static head stayed around 3.3 m on average.

This finding suggests that the wheel speed has little bearing on the pump’s maximum static head., or it doesn’t affect it. The pump speed only affects the discharge significantly.

### Effect of variation of submergence ratio

**Fig. 7:**
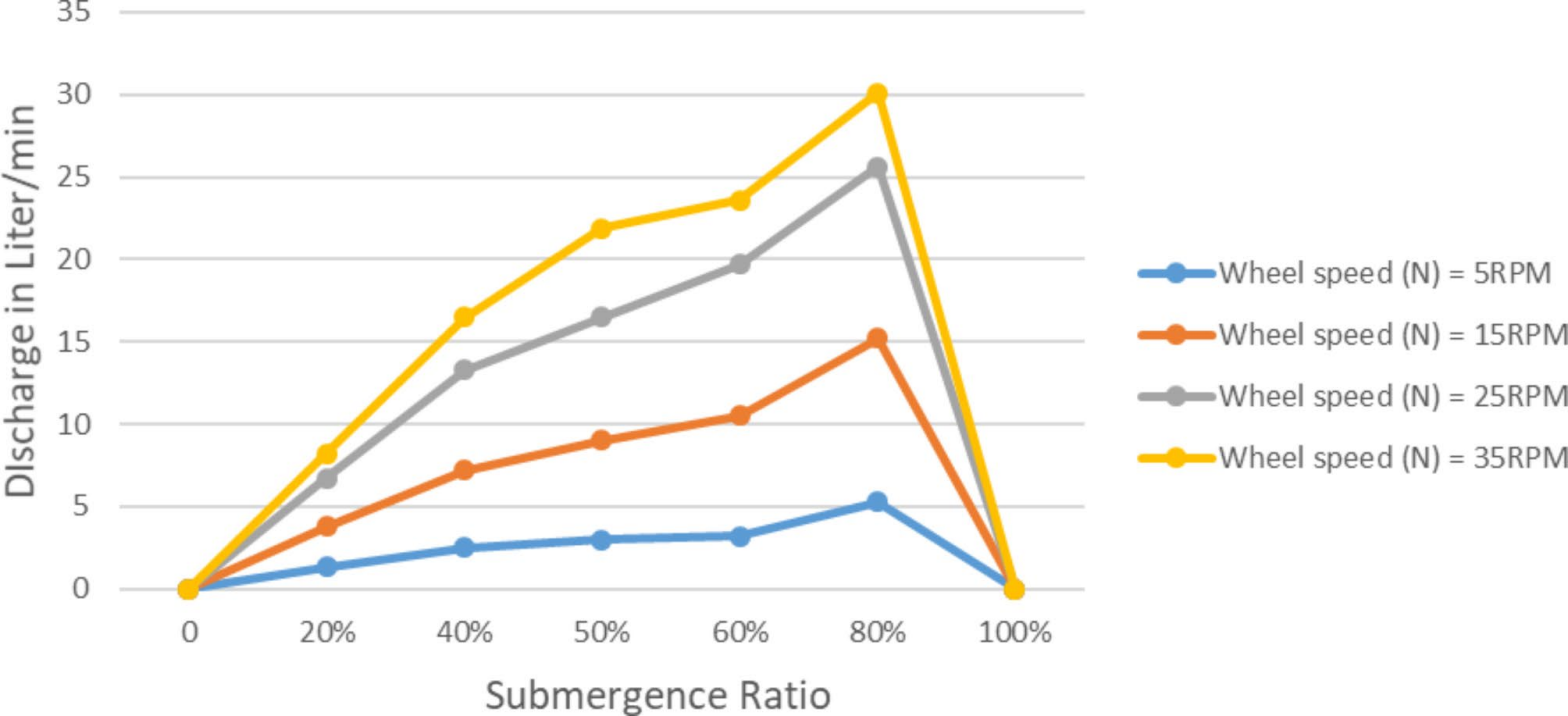
Pump discharge variation with submergence ratio at various speeds, H = 1.5 m.

**Fig. 8:**
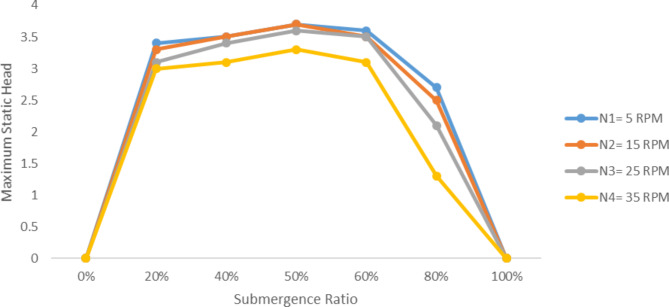
Variation of maximum static head at varying speeds with submergence ratio.

Submergence ratio is another important parameter that is labelled to have an effect on the performance of spiral. Because, it implicates the amount of water entered the spiral tube per a rotation. The discharge’s response to changes in the submerged ratio (Q) and maximum static head (H) of the pump at various wheel speed (N), is presented in Figs. 7 and 8 respectively. These findings only apply to one layer, for outer diameter, Do = 1.5 m, tube diameter, d_t_ = 3/4 in, and number of spiral turns *n* = 5 turns.

At 0% submergence there was no delivery, obviously. Since there is no inlet water there will not be output. Similarly on 100% submergence zero discharge is observed. This is because the idea of 100% submergence is in the contrary of the working principle of manometric pump. Because a manometric pump to be functional there must be a plug of air compressed between plug of water.

While a pump was rotating at 5 RPM around 1.34 lit/min discharge is recorded at 20% submergence. At same speed the discharge raised to 5.3 lit/min when the submerged 80%. The maximum discharge recorded was 30 lit/min.

So that when submergence ratio approaches to 100% the discharge of the Spiral Tube Pump increase until it decline and upon complete submersion of the pump, return to zero. The submergence ratio is directly proportional with pump discharge.

Unlike pump discharge the maximum static head exhibits insignificant increase with the increase of submergence ratio. It is obvious the head is zero at 0% and 100% submergence ratio. At 5 RPM wheel speed the pump head was around 3.5 m up to about 60% submergence ratio, though around 80% the head declined to 2.8 m. This is implies the spiral tube was unable to let enough air mass. So that the cumulative head build up lessened. In general variation in submergence ratio has negligible effect on maximum static head for wider range of submergence ratio. Though when the submergence ratio get closer to 100% it affects the maximum head noticeably.

So both the speed of the wheel and submergence ratio is directly proportional with pump discharge, and has insignificant effect on the maximum static head. This implies the theoretical relationship by Mortimer on Eq. 2.11 to be true for spiral type manometric pumps as of coil pumps.

### Effect of variation of number of spiral turns

**Fig. 9:**
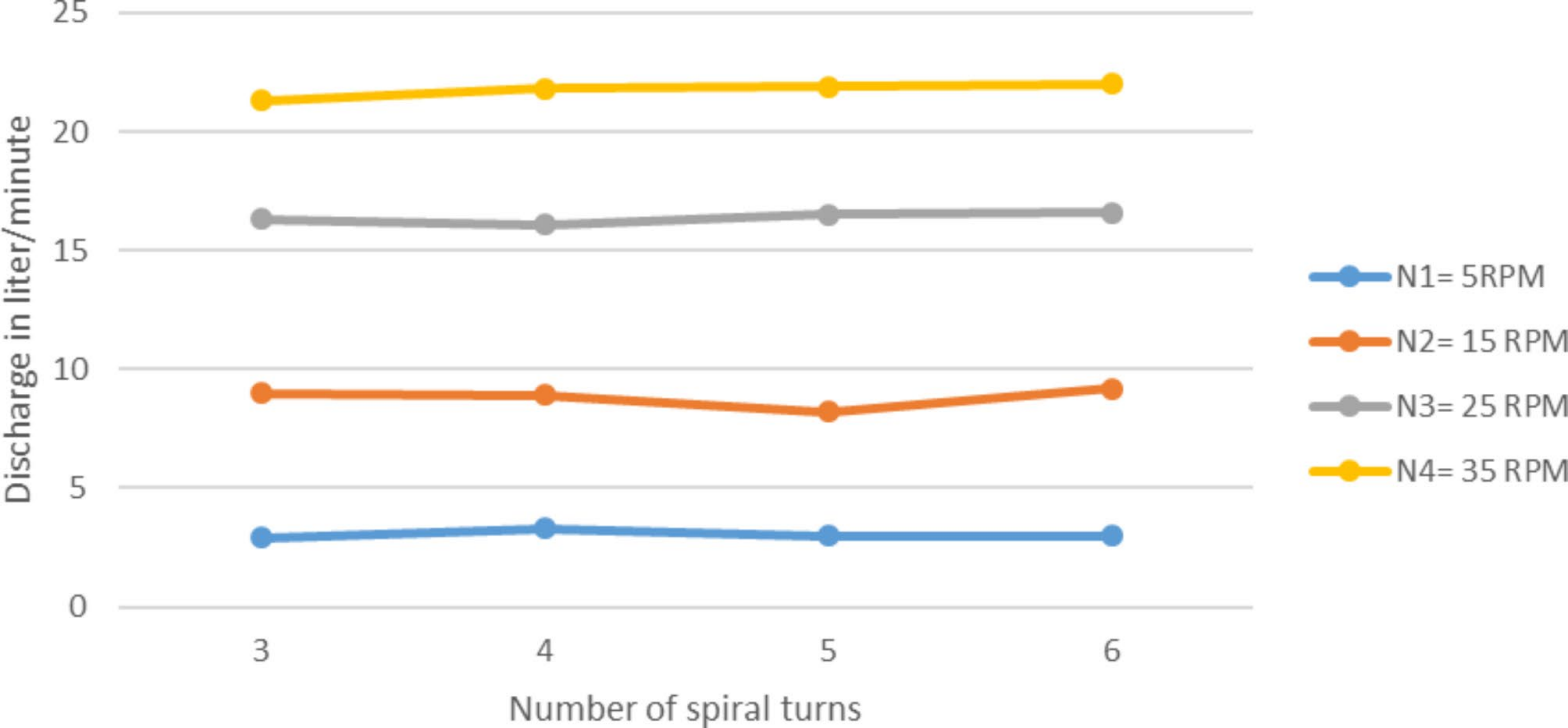
Variation of pump discharge with number of spiral turns in at different speed, H = 1.5 m.

Number spiral turns (n) variation also depicted an important change on the performance of pump. In case of discharge of the effect was not noticeable, even though the increase in number of spiral turns affected the pump maximum head enormously. Experiment results for one layer, for outer diameter, Do = 1.5 m, tube diameter, d_t_ = 3/4 in, submergence ratio 50% for varying spiral turns and with altering wheel speed is presented on Fig. 9.

For 5 RPM wheel speed the pump discharge stayed to be around 33 lit/min while the spiral turn number increase from 3 to 6. This result was expected because it is predicted that the number of spiral turns to have no effect on the amount of water induced to the pump or the number of times a certain amount of water induced to the pump system. While conducting the experiment the diameter of outer or the last turn kept unchanged by adjusting the inner turns to let increment in number. Variation of pump discharge with number of spiral turns in at different speed, H = 1.5 m is shown in Fig. 9.

**Fig. 10:**
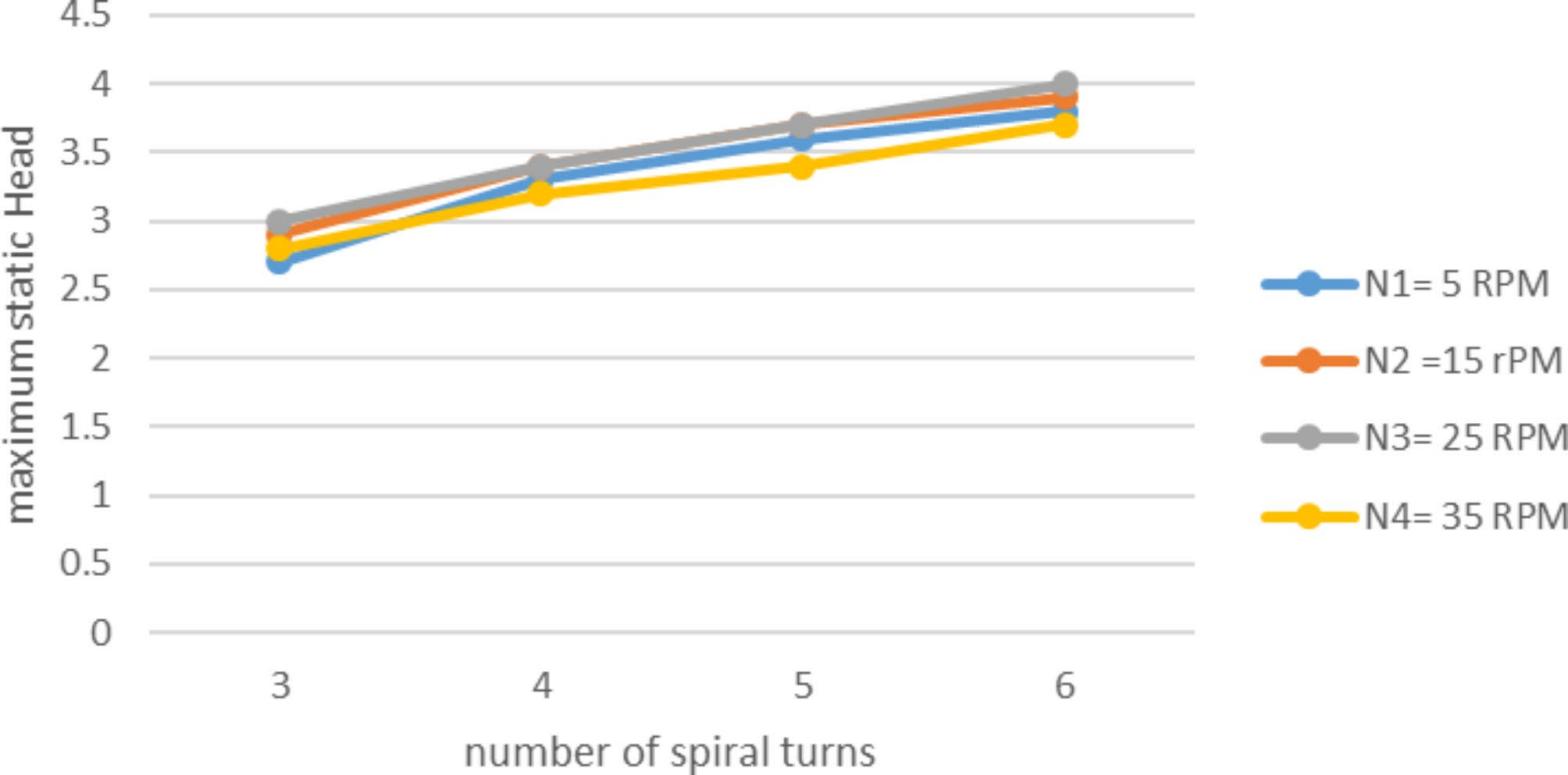
variation of max. Static head with number of spiral turns in at different speed.

Unlikely the variation in the number of spiral turns altered the pump maximum static head vitally. As illustrated in Fig. 10 the increment on number of turns from 3 to 6 raised the head from 2.7 m to about 3.8 m. If it wasn’t for the leakage the maximum static head would be about 25% higher. This means the pump static head is a function of number of the spiral turns of the tube as Naegel reported. This result coincides with the principle of head development on the manometric pump. Accordingly head is developed in cascaded manometric principle. So that if there is higher number of spiral turns there will be higher number of cascaded manometers as a result higher cumulative headed will be developed.

In conducting this experiment diameter of each turn in the spiral is crucial. To generate the same result as represented on Fig. 10. It is important to set the diameter of each turn as stated in Table 2.

**Table 2:** Diameter of each turns of spiral tube.

Number of spiral turns	Diameter of spiral turns from outer to inner
3	1.5, 1.2, 1
4	1.5, 1.2, 1, 0.8
5	1.5, 1.2, 1, 0.8, 0.6
6	1.5, 1.2, 1, 0.8, 0.6, 0.4

### Effect of outer diameter

**Fig. 11:**
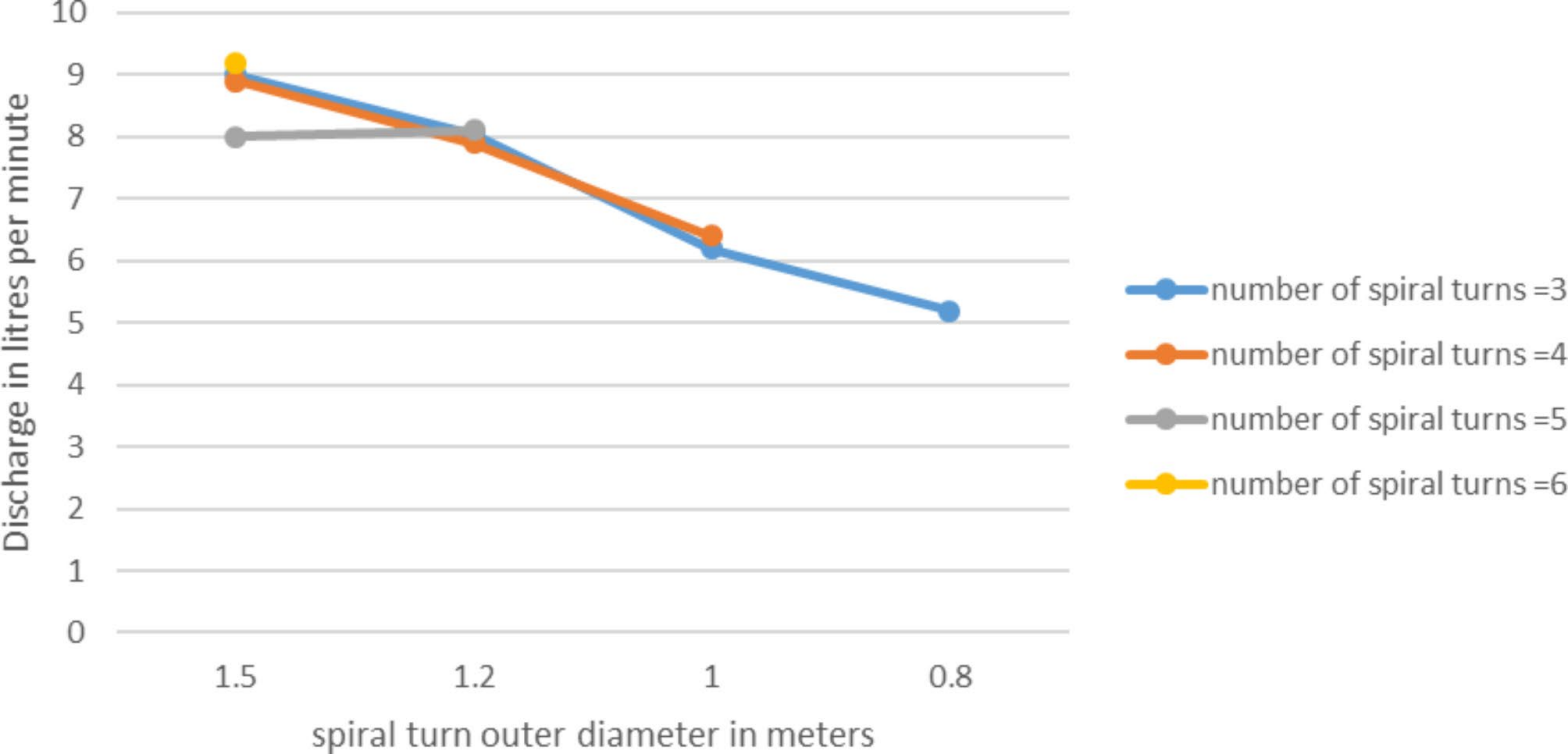
Variation of pump discharge with outer diameter for different number of spiral turns, H = 1.5 m.

**Fig. 12:**
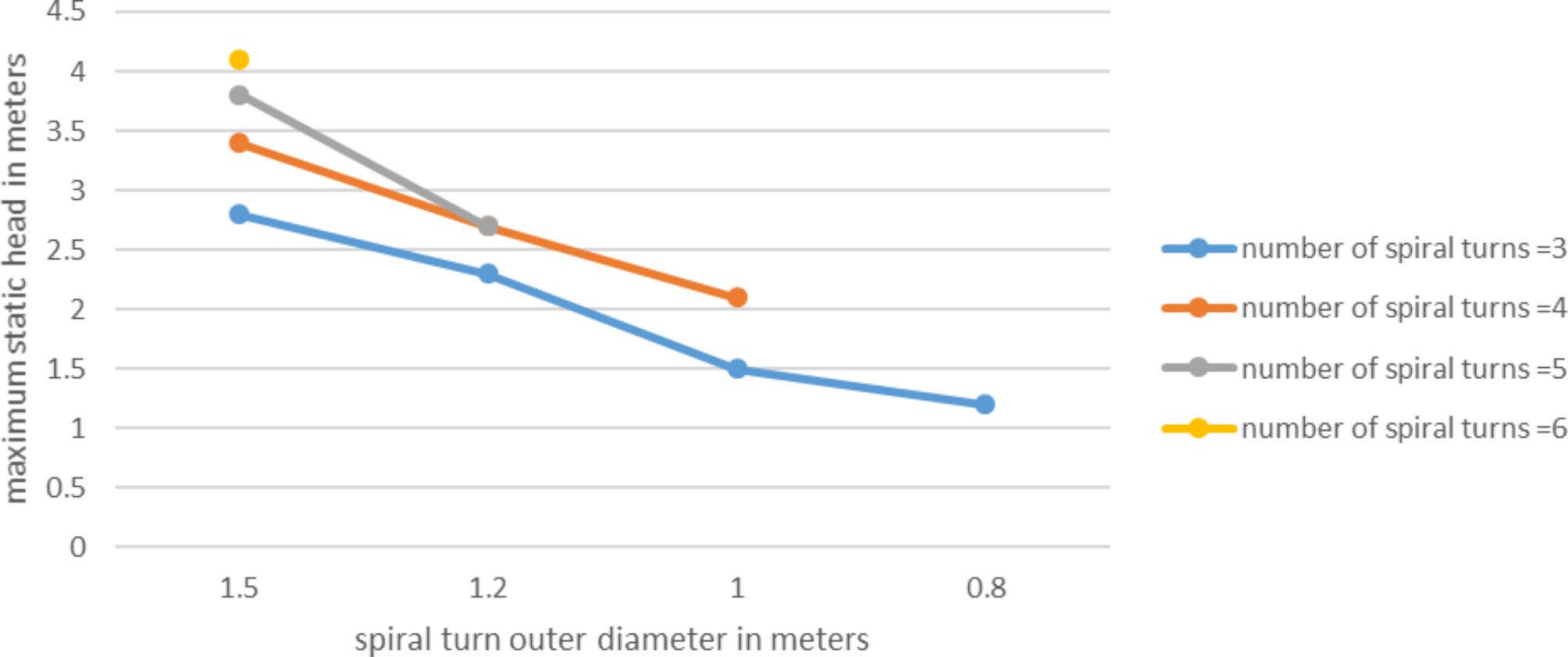
Variation of pump max. Static head with outer diameter for different number of spiral turns.

Outer diameter is an important concept that differentiate spiral tube pump from coil pump. In spiral tubes for each spiral turn increase there will be an increment on the outer diameter. On the contrary in case of coil pumps the outer diameter is always constant. The tube is coiled around a constant diameter cylinder or drum. Based on this understanding the experiment was conducted with 15 RPM speed, at Sr = 50%. According to result shown on Figs. 11 and 12 both the pump discharge and the head has shown increase with the increase of outer diameter.

The maximum static head increased for the increase of outer diameter. At outer diameter of 0.8 m the head was 1.2 m with 3 number of spiral turns, when the outer diameter get wider to 1.5 the maximum static head elevated to about 3 m, this is significant. This can be related with the concept of cascaded manometers. The wider diameter refers to larger manometers in the manometers series. So larger manometer results larger head per turn.

Therefore, the outer diameter is the one parameter that affects both pump discharge and head significantly.

### Validation of experimental results with theoretical relations

**Fig. 13:**
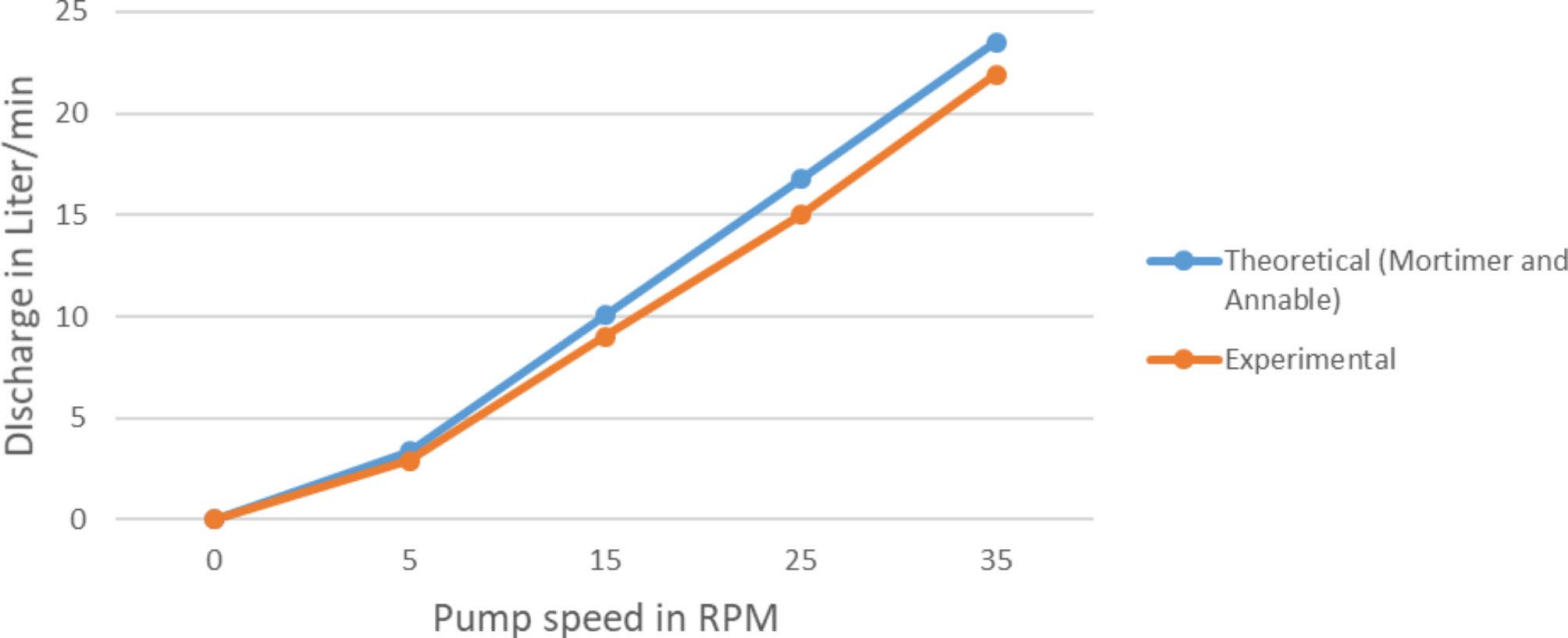
Comparison of the real and theoretical pump discharge at various rotating speeds.

**Fig. 14:**
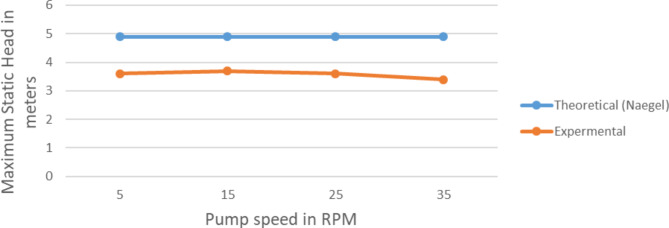
A comparison of the experimental and theoretical Pump max values. Static head rotating at various speeds.

The Mortimer^3^ and Naegel^2^ had clearly stated the theoretical relation between the important parameters and pump discharge and pump head. In this section the theoretical output prediction based on Eqs. 3 and 5 is compared with the field experiment result and presented on the Figs. 13 and 14.

Pump discharge result of the experiment values deviates from the analytical by about 8% error considering the condition of experimenting this value is very less. So it can be concluded the experimental result is validated by the theoretical prediction.

In case of the head 25% error is exhibited. This error because of the air leakage at the rotary joint. So when we consider this situation the error will be acceptable. So here also the experimental result is also validated.

## Conclusions

The Spiral Tube Pump, along with other similar manometric pumps, presents a promising solution for low-cost, emission-free, and straightforward irrigation pump needs. By integrating the Spiral Tube Pump with water wheels, it can harness the kinetic energy of rivers, requiring no additional energy costs. The performance of the Spiral Tube Pump is influenced by key factors, including wheel speed, submergence ratio, the number of spiral turns, and the outer diameter of the spiral. The observed effects of these parameters can be summarized as follows:

- The wheel speed and submergence ratio have a significant impact on the pump’s discharge. For 60% wheel speed increase up to about 500% discharge increase was observed. Also for 300% increase in submergence ratio up to about 275% increase in discharge was observed. However for the same amount increase in both wheel speed and submergence ratio the observed variation in head was insignificant.
- The number of spiral turns primarily influences the pump’s head. For 100% increase in spiral turn up to about 33% increment in head was observed. However the variation in discharge was insignificant.
- The outer diameter affects both the pump’s discharge and head. For 87% increase in outer diameter up to about 80% of increase in discharge and up to about 163% increase in head was observed.

These findings suggest that the design of a Spiral Tube Pump can be tailored to meet specific irrigation field conditions. For example, in situations where the irrigation field is situated at an elevated position relative to the river, incorporating a higher number of spiral tube turns can enable the pump to effectively transport water to the desired location.

For the future, the potential for developing a numerical approach to study the behavior and performance of the Spiral Tube Pump holds promise. Such an approach could lead to optimized designs and a deeper understanding of the pump’s dynamics, making it even more suitable for irrigation purposes in the future.

## Data Availability

All data generated or analysed during this study are included in this published article.
